# The use of cyproterone acetate in a forensic psychiatric cohort of male sex offenders and its associations with sexual activity and sexual functioning

**DOI:** 10.4102/sajpsychiatry.v23i0.982

**Published:** 2017-03-10

**Authors:** Gian Lippi, Paul J. van Staden

**Affiliations:** 1 Department of Psychiatry, Faculty of Health Sciences, University of Pretoria, South Africa; 2 Department of Statistics, Faculty of Natural and Agricultural Sciences, University of Pretoria, South Africa; 3 Forensic Unit, Weskoppies Hospital, South Africa

## Abstract

**Background:**

Cyproterone acetate (CPA) is a steroidal anti-androgenic medication used in the field of psychiatry for the treatment of paraphilic disorders, hypersexuality, and inappropriate sexual behaviour which may be present in patients with disorders such as mild and major neurocognitive disorders. In the forensic psychiatric population, it is prescribed for these indications especially for patients with a history of committing a sexual offence or who are at moderate to high risk of recidivism.

**Objectives:**

To investigate the use of CPA in a forensic psychiatric cohort of male sex offenders and its associations with sexual activity and sexual functioning.

**Methods:**

Seventy-six forensic psychiatric patients from Weskoppies Hospital in Pretoria, South Africa, participated in the study which measured their sexual functioning. A specifically designed questionnaire was used to capture relevant background information. The use of CPA was studied. The Changes in Sexual Functioning Questionnaire, Male Clinical Version (CSFQ-M-C) was used to measure sexual functioning of participants. The CSFQ-M-C scores, and those of all its subscales, of participants on CPA were compared to those not on the drug. Relevant statistical analyses were performed.

**Results:**

Thirteen out of the 76 participants were being treated with CPA (17.11%). In total, 53.85% of the participants on CPA and 65.08% not on CPA had scores indicating the presence of sexual dysfunction. The total CSFQ-M-C scores for participants on CPA (mean = 40.54; median = 42) were not statistically significantly lower than those not on the drug (mean = 41.22; median = 41). More notable is that the use of CPA in this population was associated with lower levels of desire, frequency of and pleasure from sexual activity. There was an association between having intellectual disability and being treated with CPA.

**Conclusion:**

That all the participants were being treated with psychotropic medication could account for the high percentage of sexual dysfunction in any or all areas of sexual functioning and contribute to the small difference in CSFQ-M-C scores between the two groups. Only a tentative conclusion can be made that CPA may be more effective in decreasing levels of desire, frequency and pleasure related to sexual activity than other areas of sexual functioning. The indication for the use of CPA in this population should be assessed clinically according to patient circumstances and risk assessment.

## Introduction

Cyproterone acetate (CPA) is a steroidal anti-androgenic medication used in the field of psychiatry for the treatment of hypersexuality and inappropriate sexual behaviour.^1,2,3^ It acts through binding as a competitive inhibitor of testosterone (T) and dihydrotestosterone at all types of androgen receptor sites, including those in the brain, blocking intracellular T uptake and metabolism. CPA also has a strong progestogen action that causes inhibition of gonadotropin-releasing hormone (GnRH) secretion from the hypothalamus, resulting in a decrease of both luteinising hormone and follicle-stimulating hormone release from the anterior pituitary.^2,3,4,5,6,7,8,9^

CPA is prescribed in the forensic psychiatric population for sexually inappropriate behaviour, especially for patients with a history of committing a sexual offence or who are at moderate to high risk of recidivism.^1,5^ The decision to prescribe the medication is often based not exclusively on sexual offence histories or risk profiles but also on specifics of patient profiles including diagnoses and symptomatology.^2,3,4,5,6,7,8,9,10,11,12,13,14^ Its diagnosis-guided use is usually in the context of treatment of paraphilic disorders where it is recommended for use in more severe cases, for patients with a ‘hands on’ disorder like paedophilic disorder who have a history, or are at risk of acting on their urges, or for milder cases where psychotherapy and first-line pharmacotherapy agents have been ineffective.^2,3,4,5,6,7,9,14^ Paraphilic disorders, because of their very nature, can lead to behaviour which is illegal. Treatment of these disorders is often, but not necessarily, in the forensic psychiatric context. Both general and forensic psychiatric treatment settings are also possible for the management of patients with intellectual disability (ID) who present with hypersexuality or inappropriate sexual behaviour. The use of CPA to attempt to manage this problematic behaviour in this subpopulation can be advocated in patient-specific appropriate situations.^8^ The medication has also shown efficacy in the management of similar hypersexual behaviours in mild and major neurocognitive disorders in the general psychiatric treatment setting.^10,12,13^ The use of CPA in the general psychiatric population has been largely limited by its side effect profile.^1^ One of the deductions one can make from this finding is that its positive effects may well be outweighed by the negative effects of its side effect profile within the general psychiatric population.

There are testaments to its efficacy in the literature, with decreased sexual drive, urges, fantasies, arousal, activities and behaviours, as well as reduced erections and fewer orgasms being reported,^2,3,4,5,6,7,8,10,11,12,13,14,15,16,17,18^ yet it is not uncommon to find opinions from clinicians questioning its use in the context of the potentially serious side effects of androgen-deprivation treatment.^16,17,19,20,21^ For a list of potential side effects of CPA, including those related to androgen deprivation, refer to Table 1. The concerns extend to its use even in the forensic psychiatric population especially in the light of increasing research into the efficacy and use of selective serotonin reuptake inhibitors (SSRIs) which are more widely used in psychiatry and offer, arguably, a more ethical treatment alternative in the forensic psychiatric population.^1^

**TABLE 1:** Possible side effects of cyproterone acetate.

Side effects related to androgen deprivation	Other possible side effects of cyproterone acetate
Osteoporosis Hair loss or pilosity changes Dry skin Epidermal thinning Stria and skin discolouration Decreased sebum ejection rate Feminisation and hypogonadism Hot flashes or cold sweats Asthenia or fatigue Sleep disorders Depressive symptoms or disorders Emotional disturbances Leg cramps and decreased muscle mass Decreased spermatogenesis Infertility Decreased ejaculate volume Reduced libido and sexual fantasies† Impotence or erectile dysfunction† Decreased sexual activity† Decreased serum testosterone†	Weight gain Pituitary dysfunction Increased prolactin levels Gynaecomastia and mastodynia Galactorrhoea Liver dysfunction Hepatocellular damage Hepatic cirrhosis Hepatocellular carcinoma Carcinogenic effects Small increases in serum bilirubin Adrenal insufficiency or hyperplasia Deep vein thrombosis and thrombo-embolic phenomena Varicose veins Nausea, vomiting and constipation Headache and neurasthenia Dyspnoea and ischaemia Hypertension or blood pressure changes Anaemia and cardiac insufficiency Decreased glucose tolerance or diabetes mellitus Kidney dysfunction Dyspepsia and gallstones Nightmares Benign cerebral meningioma Negative nitrogen balance (temporary) Pain at injection site (depot formulation) Prevents onset of puberty and affects epiphyseal closure in children

† , Possible treatment aims in patient-specific sexual offenders.

Very few studies have been published on the efficacy of CPA or other androgen-depleting medications like medroxyprogesterone acetate (MPA) or the GnRH analogues [also known as GnRH agonists or luteinising hormone-releasing hormone (LHRH) agonists] like leuprolide, triptorelin and goserelin in the treatment of hypersexuality and sexually inappropriate behaviour, and high-quality evidence in favour of their use is lacking.^4,8,10,11,12,15,18^ There have been no randomised control trials on the subject in the last two decades,^18^ which is a good reason for a study to be conducted where the use and effects of CPA are evaluated. Most published studies report on CPA use in the treatment of both paraphilic disorders and sexual offenders.^2,3,4,5,6,9,14,18^ Systematic reviews and meta-analyses have come to differing conclusions as to the efficacy of CPA, with some concluding that there is enough evidence to recommend its use and others that the evidence is not strong enough to recommend it for the treatment of paraphilic disorders, hypersexuality and inappropriate sexual behaviour.^2,4,5,7,8,10,11,12,13,14,18^ Many studies used recidivism rates as the outcome measure.^3,4,8,14,18^ Others also report on multiple outcome measures, including reductions in sexual behaviours and deviant sexual fantasies.^2,3,4,5,6,7,8,18^ In these studies, sexual drive, functioning and behaviours were mostly measured secondary to what was reported by the patients themselves or by family members or caregivers and included investigating the frequency of sexual behaviours like masturbation.^2,3,4,6,10,18^

## Aims

The aims of the study were to evaluate the use of CPA in the treatment of male sex offenders at Weskoppies Hospital, to evaluate the sexual activity and sexual functioning of these patients and to compare the results with those of patients not on CPA and with published norms.

During the course of the study, 144 male sex offenders were being treated at the forensic unit of Weskoppies Hospital as State Patients in terms of Section 42 of the *Mental Health Care Act*, 17 of 2002,^22^ some of whom were on CPA treatment for sexually inappropriate behaviour in the context of their evaluated increased risk for recidivism.

## Method

A vulnerable patient population of State Patients had to constitute the study population because the aim was to evaluate the use of CPA in a psychiatric population of male sex offenders and its associations with sexual activity and sexual functioning. The study was approved by the Faculty of Health Sciences’ Research Ethics Committee of the University of Pretoria. During the informed consent process, patients were informed about their right to choose whether or not they wanted to participate in the study and that their choice would not affect the care, treatment and rehabilitation they received and also would not affect decisions surrounding their legal status as State Patients.

Compared to studies previously mentioned,^2,3,4,6,10,18^ a more structured method of measurement of sexual functioning was used in the current study in the form of a rating scale, the Changes in Sexual Functioning Questionnaire, Male Clinical Version (CSFQ-M-C).

**FIGURE 1:**
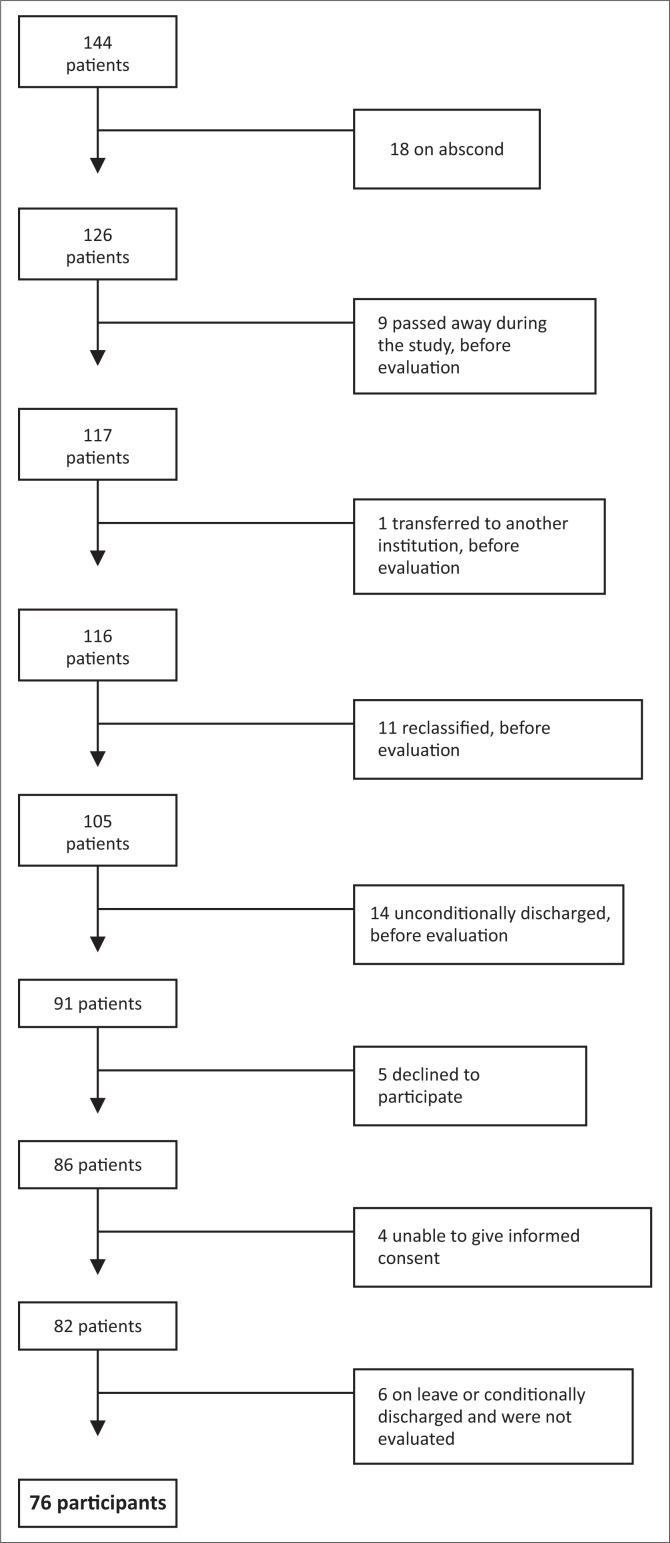
Participant recruitment.

From the 144 patients, 76 constituted the study population. Reasons for not including the remaining 68 patients are provided in Figure 1.

The CSFQ-M-C was administered to the participants to measure their sexual functioning. It measures overall sexual functioning and consists of subscales that measure different aspects of sexual functioning (i.e. sexual desire, interest and pleasure, as well as arousal, erection, orgasm or ejaculation and frequency of sexual activity).

A specifically designed data capture sheet was used to collect background information of the participants from their clinical files. Information captured was about whether patients were on CPA, and if so, dosages and length of time on treatment. Information on the concomitant use of MPA with its dosages was collected as well as on age, diagnosis, the offence committed and the treatment setting (i.e. closed ward, open ward or outpatient).

Data of the participants on CPA, including their CSFQ-M-C scores and those of all its subscales, were compared to those of participants who had not been on CPA, the latter thus serving as the non-matched control group.

## Statistical analysis

The statistical data analysis was done using SAS software, Version 9.3 of the SAS System for Windows. Copyright © 2002–2010 SAS Institute Inc. SAS and all other SAS Institute Inc. product or service names are registered trademarks or trademarks of SAS Institute Inc., Cary, NC, USA. Means and standard deviations were calculated for the CSFQ-M-C total and subscale scores. Quartiles, including medians, and minimum and maximum values were calculated for these scores, and boxplots were drawn for comparing the scores of the groups. Mann–Whitney *U* tests were used to test whether the scores of participants being treated with CPA are significantly different than the corresponding scores of participants not on CPA treatment.

For categorical variables, frequencies and percentages were calculated. The chi-square test for independence and Fisher’s exact test in cases of low expected frequencies were used to test for statistical association between CPA treatment and various clinical and demographic variables, which included the presence of sexual dysfunction, offence committed, treatment setting and diagnoses of disorders. For major depressive disorder (MDD), a binomial test was used to compare the prevalence of this disorder in the sample of participants with the lifetime prevalence in South Africa reported elsewhere.^23^

## Results

**FIGURE 2:**
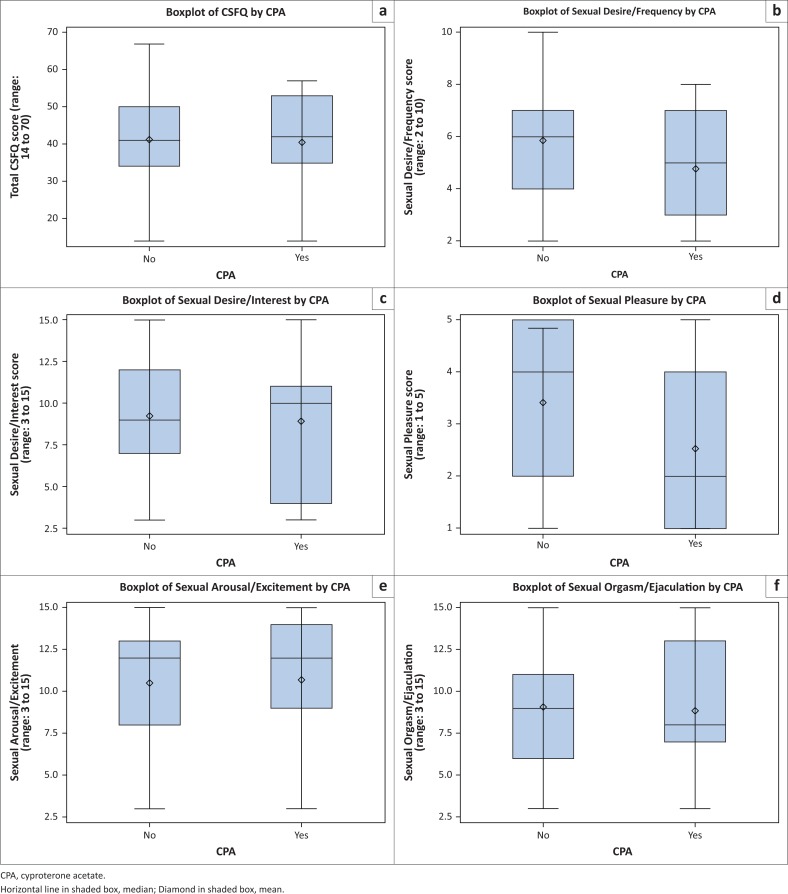
Boxplots of CSFQ-M-C total and subscale scores.

Thirteen of the 76 participants were being treated with CPA (17.11%). In total, 53.85% (7 of 13) of the participants on CPA and 65.08 % (41 of 63) not on CPA had scores indicating the presence of sexual dysfunction (*p* = 0.4446, signifying no statistical dependence between CPA treatment and the presence of sexual dysfunction). The total CSFQ-M-C scores for participants on CPA (mean = 40.54; median = 42; standard deviation (SD) = 15.50) were not statistically significantly lower than those not on the drug (mean = 41.22; median = 41; SD = 11.45), *p* = 0.397. The highest total CSFQ-M-C score for participants on CPA was 57, compared to 67 in the group not on CPA, of whom four scored more than 57 (the maximum in the group on CPA). Regarding the total CSFQ-M-C scores and those of the subscales, for participants both on and not on CPA, refer to Figure 2 for boxplots and to Table 2 for summary statistics.

**TABLE 2:** Summary statistics of CSFQ-M-C total and subscale scores.

Variable	Numbers	Scale or subscale	Median	Mean	s.d.	Minimum	Maximum
Not on CPA	63	Pleasure	4.00	3.41	1.58	1.00	5.00
		Desire or frequency	6.00	5.84	2.14	2.00	10.00
		Desire or interest	9.00	9.24	3.09	3.00	15.00
		Arousal or erection	12.00	10.51	3.84	3.00	15.00
		Orgasm or ejaculation	9.00	9.06	3.40	3.00	15.00
		Total CSFQ-M-C	41.00	41.22	11.45	14.00	67.00
On CPA	13	Pleasure	2.00	2.54	1.78	1.00	5.00
		Desire or frequency	5.00	4.77	2.09	2.00	8.00
		Desire or interest	10.00	8.92	4.25	3.00	15.00
		Arousal or erection	12.00	10.69	4.19	3.00	15.00
		Orgasm or ejaculation	8.00	8.85	4.38	3.00	15.00
		Total CSFQ-M-C	42.00	40.54	15.50	14.00	57.00

s.d., standard deviation.

Regarding the subscales of (1) desire or interest, (2) arousal or erection and (3) orgasm or ejaculation, the scores of participants on CPA were also not statistically significantly lower than the scores of participants not on CPA. However, at a 5% significance level, the scores for the subscale of pleasure for participants on CPA (mean = 2.54; median = 2) were significantly lower than those not on the medication (mean = 3.41; median = 4), *p* = 0.030.

Similarly, at a 10% significance level, the scores for the subscale of desire related to frequency of sexual activity for participants on CPA (mean = 4.77; median = 5) were significantly lower than those who are not (mean = 5.84; median = 6), *p* = 0.065.

For a summary of the *p*-values of the Mann–Whitney *U* tests for comparing the total CSFQ-M-C scores and the subscale scores refer to Table 3.

**TABLE 3:** Mann-Whitney *U* test *p*-values for testing whether the total CSFQ-M-C score and the subscale scores are significantly lower for participants on CPA than for participants not on CPA.

Scale or subscale	*p*
Pleasure	0.0298*
Desire or frequency	0.0653**
Desire or interest	0.4531
Arousal or erection	0.4094
Orgasm or ejaculation	0.4366

**Total CSFQ-M-C**	**0.3966**

CPA, cyproterone acetate.

* , 5% significance level;

** , 10% significance level.

High numbers and percentages of participants both on and not on CPA presented with CSFQ-M-C subscale scores indicative of the presence of sexual dysfunction in the various areas of sexual functioning (Table 4), but no statistically significant association was found between CPA treatment and the presence of sexual dysfunction in the various areas.

**TABLE 4:** Numbers and percentages of participants on and not on CPA that have CSFQ-M-C subscale scores which are indicative of the presence of sexual dysfunction in a particular area of sexual functioning.

Subscale	Dysfunctional	Not on CPA	On CPA
Pleasure	score ≤ 4	57.14% (*n* = 36)	76.92% (*n* = 10)
Desire or frequency	score ≤ 8	92.06% (*n* = 58)	100.00% (n = 13)
Desire or interest	score ≤ 11	73.06% (*n* = 46)	76.92% (*n* = 10)
Arousal or erection	score ≤ 13	76.19% (*n* = 48)	69.23% (*n* = 9)
Orgasm or ejaculation	score ≤ 13	90.48% (*n* = 57)	84.62% (*n* = 11)

CPA, cyproterone acetate.

Of the 13 participants on CPA, only two were on a monthly intramuscular dose of 150 mg, while 11 were on 300 mg. Except for the subscale of pleasure, all the other subscale scores as well as that of the total CSFQ-M-C were lower for those on 300 mg than those on 150 mg. These differences are not statistically significant.

No participant had been on CPA for longer than 5 years. Ten had been on the drug for between 1 and 5 years and three for less than a year. Except for the subscale of pleasure, all the other subscale scores as well as that of the total CSFQ-M-C were lower for those who had been on the drug longer. Again, these differences are not statistically significant. Only one participant was also being treated with MPA, and he was on a monthly dose of 150 mg.

The mean age of the participants was 39.26 (SD = 10.05), the youngest being 22 and the oldest 69. The mean ages of the two groups were very similar (mean = 39.77; SD = 13.29 for those on CPA vs. mean = 39.16; SD = 9.38 for those not on CPA) indicating no association between age and being treated with CPA.

Fifty-one participants had been diagnosed with schizophrenia (67.11%), one with schizoaffective disorder (1.32%), 14 with unspecified schizophrenia spectrum disorder (18.41%), one with MDD (1.32%), three with bipolar disorder (3.95%), 13 with ID (17.11%), three with antisocial personality disorder (ASPD) (3.95%), two with personality change due to another medical condition (2.63%), two with paedophilic disorder (2.63%) and one each with exhibitionistic disorder and voyeuristic disorder (1.32%).

**FIGURE 3:**
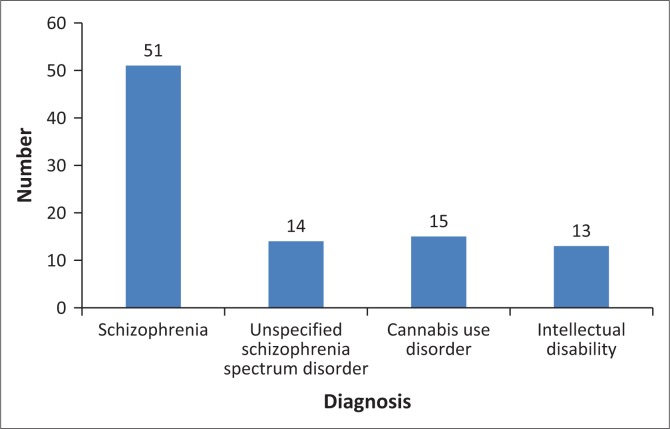
Most common diagnoses in the participant population.

Cannabis use disorder had been diagnosed in 15 participants (19.74%) and alcohol use disorder in four (5.26%). Two participants were using multiple substances (2.63%). For graphic representation of the most common diagnoses in the population refer to Figure 3.

There was an association at a 1% significance level of having ID and being treated with CPA (*p* = 0.007). No association was found between having any other diagnosis and receiving CPA treatment.

Rape was the sexual offence committed by 67 participants (88.16%). Three participants committed attempted rape (3.95%) and seven committed indecent or sexual assault (9.21%). There was no association between having committed a specific sexual offence and being treated with CPA.

Of the 63 participants not on CPA, 41 were being treated in a closed or semi-closed ward (65.08%), 13 in an open ward (20.63%) and 9 as outpatients (14.29%). Ten of the 13 participants on CPA were being treated in a closed or semi-closed ward (76.92%), 2 in open wards (15.39%) and 1 was an outpatient (7.69%). No association was found between being on CPA and being treated in any specific ward.

## Discussion

The fact that 17.11% of the study population of sex offenders were being treated with CPA is similar to the findings of two studies involving German forensic psychiatric hospitals and outpatient clinics where 12% (in the 2002 study) and 15.7% (in the 2013 study) of the sex offenders received either CPA or LHRH agonists.^5,7,16,18^ In the United States in 2009, depending on the setting and type of drug, between 13% and 17% of patients received androgen-deprivation drugs. In Canada, these numbers varied more widely with a minimum of 17% and a maximum of 75% of patients receiving androgen-deprivation drugs.^7^ The similarity of prescribing habits of CPA at Weskoppies Hospital compared to patients in equivalent populations in Germany, the United States and Canada and the fact that in our study there was no association between being on CPA and (1) age, (2) the treatment setting (i.e. closed ward, open ward or outpatient), (3) having committed a specific sexual offence or (4) having a specific diagnosis (ID excluded) suggest the responsible use of CPA in the treatment of sex offenders at the forensic psychiatry unit in that it is not being prescribed injudiciously in this high-risk patient population.

The association found between having ID and being treated with CPA may be due to assessed risk of recidivism according to (1) a history of a sexual offence, (2) the presence of hypersexuality of sexually inappropriate behaviour in the hospital and (3) the fixed nature of the perceived inability of many of the patients to appreciate the wrongfulness of their sexual behaviour. Furthermore, some of these patients with ID have no comorbid psychiatric diagnosis and are therefore not receiving treatment with antipsychotics or antidepressants that may have an effect on their sexual functioning. The reliability of adherence to prescribed medication of these patients once back in the community may account for prescribing depot CPA as the treatment of choice for hypersexuality or inappropriate sexual behaviour above other treatment options like SSRIs. Owing to the fact that men with ID are twice as likely to commit sexual offences as those without and that their recidivism rates are also higher, the increased incidence of CPA treatment in this subpopulation is understandable. In people with ID, the choice of treatment should depend on individual circumstances which are unique and include the severity of ID, the individual’s capacity to give informed consent to treatment, availability of resources to monitor treatment, legal status and the risk posed to themselves and others.^8^

The low number of participants with a diagnosis of either ASPD or one of the paraphilic disorders is surprising. It is likely that these conditions are being underdiagnosed in this population, probably because most of the participants would have had another, comorbid, primary, more obvious diagnosis like schizophrenia, which would explain why they were in the forensic psychiatric population in the first place, and not in prison, where most offenders with ASPD and paraphilic disorders as a primary diagnoses are likely to be found. Furthermore, the diagnosis of ASPD and paraphilic disorders depends a lot on the admission of symptoms and honesty of patients, both of which are likely to be lacking in patients with these disorders, especially in the forensic setting. Because of the low prevalence of MDD in the sample (1.32%), it seems that this disorder is also being underdiagnosed. This prevalence is statistically significantly less at a 5% significance level than the lifetime prevalence of MDD in South Africa of 9.7% (*p* = 0.0135).^23^

The non-association between specific offences and whether or not participants were being treated with CPA indicates that patients are not being treated with CPA according to the seriousness of the sexual offence they committed, which is a positive finding for the ethical clinical management of these patients.

The fact that Weskoppies Hospital has almost three times as many closed or semi-closed ward beds than open ward beds contextualises the findings that there are just more than three times as many study participants being treated in closed or semi-closed wards than in open wards.

The high percentage of participants in the study with sexual dysfunction in any or all of the areas of sexual functioning can be attributed to the fact that the vast majority of them were being treated with a psychotropic medication (72 out of 76; 94.74%). Sixty-nine of these participants were on an antipsychotic (90.79%), which are known to have sexual side effects, and the dopamine-reducing effects of which can reduce sexual drive.^2^ Six of them were on an SSRI (7.90%) which are well known to have sexual side effects and have documented evidence of reducing sexual and paraphilic fantasies, drive and activity; inappropriate sexual behaviour; and erectile, ejaculatory and orgasmic function in this patient population,^1,2,4,5,7,8,9,18^ and indeed in reducing inappropriate sexual behaviour in patients with major neurocognitive disorder.^11,12^ Three participants were being treated with both an antipsychotic and an SSRI (3.95%). The use of other psychotropic medications by the participants complicates the deductions one can make about the effects of CPA in this patient population. The lower percentage of participants on CPA having sexual dysfunction compared to those not on the drug may be because they presented with hypersexual behaviour, were therefore prescribed the drug, but have sexual functioning above the dysfunctional level despite being on the drug.

The total CSFQ-M-C scores of those on CPA were not statistically significantly lower than those not on it, probably also due to the influence of most patients being on psychotropic medications. While the differences of the CSFQ-M-C scores for the subscales for (1) desire or interest, (2) arousal or erection and (3) orgasm or ejaculation were also not statistically significant, those for pleasure and desire related to frequency of sexual activity were statistically significantly lower in participants on CPA, indicating a possible greater suppressing effect of the drug on desire to engage in sexual activities and pleasure experienced from sexual matters as opposed to other areas of sexual functioning – this was the most important finding of the study. The finding of decreases in overall sexual functioning; sexual desire, interest, arousal, pleasure and need to engage in sexual activities; and reductions in erections, orgasms and ejaculations are consistent with what is reported in other studies.^2,3,4,5,6,7,8,10,12,18^

The majority of patients being treated with psychotropic medications limits conclusions about the efficacy of CPA in the study population. But such is to be expected in a naturalistic study, mitigated by the fact that almost all the participants were on a psychotropic medication, meaning it can almost be seen as a ‘constant variable’. However, there is still pertinent information about the effects of CPA on the sexual functioning of the forensic psychiatric population where patients who require CPA treatment almost always have other psychiatric disorders that require psychotropic medications. Even those with only paraphilic disorders who may only require CPA treatment will not typically be found in the forensic psychiatric setting but in the general psychiatric population or in the prison system. Clinicians can therefore make informed decisions about similar patients they may have according to this study of patients who are not in an artificial setting. The resulting small sample size of participants on CPA also precluded some statistically significant results.

Even though the CSFQ-M-C is a self-report questionnaire, it was administered to the participants in this study because of difficulties surrounding high numbers of participants who couldn’t speak English or who were cognitively impaired or illiterate, making it a severely limiting factor and difficult to interpret the results when it was not used exactly as per its design.

Finally, there is evidence of efficacy of psychotherapy in the treatment and rehabilitation of sex offenders,^14,15,18^ but this was not investigated as a variable in this study.

## Conclusion

Owing to the small sample size of patients on CPA, only a tentative conclusion can be made that the results suggest the possibility of CPA being more effective in the suppression of sexual desire, and frequency and pleasure related to sexual activity but still having some effect on matters of sexual interest, arousal, orgasm and ejaculation. Because of the potential side effects of CPA, proper physical examinations, relevant laboratory tests and bone density scans need to be done before and while patients are treated with the medication. The indication for the use of CPA in the forensic psychiatric population should be assessed clinically according to patient circumstances and risk assessment.
